# Age, noise and cGMP: Pharmacological activation of soluble guanylyl cyclase (sGC) interacts with the progression of age related and noise induced hearing loss

**DOI:** 10.1186/2050-6511-16-S1-A81

**Published:** 2015-09-02

**Authors:** Lukas Rüttiger, Ksenia Varakina, Dorit Möhrle, Dan Bing, Peter Sandner, Marlies Knipper

**Affiliations:** 1 Department of Otolaryngology, Eberhard-Karls-Universität Tübingen, Germany; 2 Bayer Health Care, Global Drug Discovery—Common Mechanism Research, Wuppertal, Germany; 3 Hannover Medical School, Institute of Pharmacology, Hannover, Germany

## Background

Progressing loss of sensory function is a major problem of aging populations. In humans and animals, loss of auditory function becomes evident by increased hearing thresholds, altered sound processing, and abnormal perception of above-threshold sounds or phantom perceptions like hyperacusis and tinnitus [1,2]. Recent studies could identify the close relation between aging, loss of inner hair cell synaptic contacts, and auditory brainstem responses [3]. In a previous study we reported that pharmacological PDE5 inhibition reduces hair cell damage following auditory noise exposure indicating a protective molecular cascade mediated by cGMP signaling [4]. Candidate components of this protective cascade have been described within the last decades, however, the upstream components involved in the protective cascade (cGMP-generators) in the cochlea are still unknown. We therefore tested the protective potential of cGMP increase by pharmacological stimulation of soluble guanylyl cyclase (sGC) for age and noise induced progression of hearing deficits.

## Materials and methods

Synaptic structures, afferent synaptic contacts, and auditory fibers were studied in an animal model on young (3-9 month old) and aged (20-24 month old) rats. The hearing sensation was monitored by auditory evoked brainstem responses (ABR) and otoacoustic emissions (DPOAE) giving insight for the central and peripheral (hair cell function) auditory capacity, respectively. Hyperacusis and tinnitus sensation in the rat were tested using a behavioral approach [5]. We pharmacologically stimulated cGMP production by an sGC-stimulating drug administered in the food for 25 days or several months. To estimate for the number of afferent contacts CtBP2/RIBEYE immuno-positive staining at the IHC synapse was quantified.

## Results

Auditory thresholds were increased and the auditory response range was reduced over age. The characteristic changes over age were similar to the changes observed after exposure to traumatizing sound. sGC treatment interacts with the loss of age related and auditory trauma induced loss of hair cell synaptic ribbons in a complex way, proposing that a subclass of auditory fibers with a special vulnerability can be rescued by sGC treatment within a defined time window.

## Conclusion

Age related and noise induced decline of hearing function are correlated with auditory responses and morphological specifications of hair cell molecular phenotype. The data need to be discussed regarding the proposed cGMP generators in the inner ear and their role for an otoprotective molecular cascade after noise induced damage of the ear or progression of presbycusia.
